# Treatment of Advanced Melanoma: Past, Present and Future

**DOI:** 10.3390/life10090208

**Published:** 2020-09-16

**Authors:** Taku Fujimura, Yumi Kambayashi, Kentaro Ohuchi, Yusuke Muto, Setsuya Aiba

**Affiliations:** Department of Dermatology, Tohoku University Graduate School of Medicine, 1-1 Seiryo-machi, Aoba-ku, Sendai 980-8574, Japan; yumi1001@hosp.tohoku.ac (Y.K.); r2v7ib@bma.biglobe.ne.jp (K.O.); yusuk0610@derma.med.tohoku.ac.jp (Y.M.); saiba@med.tohoku.ac.jp (S.A.)

**Keywords:** metastatic melanoma, BRAF inhibitors, MEK inhibitors, immune checkpoints inhibitors, combination therapy

## Abstract

Therapeutic options for treating advanced melanoma are progressing rapidly. Until six years ago, the regimen for treating advanced melanoma mainly comprised cytotoxic agents such as dacarbazine, and type I interferons. Since 2014, anti-programmed cell death 1 (PD1) antibodies have become recognized as anchor drugs for treating advanced melanoma with or without additional combination drugs such as ipilimumab. In addition, v-Raf murine sarcoma viral oncogene homolog B1 (BRAF) kinase inhibitors in combination with mitogen-activated protein kinase kinase (MEK) inhibitors are among the most promising chemotherapeutic regimens for treating advanced BRAF-mutant melanoma, especially in patients with low tumor burden. Since anti-PD1 antibodies are widely applicable for the treatment of both *BRAF* wild-type and mutated advanced melanomas, several clinical trials for drugs in combination with anti-PD1 antibodies are ongoing. This review focuses on the development of the anti-melanoma therapies available today, and discusses the clinical trials of novel regimens for the treatment of advanced melanoma.

## 1. Introduction

Until 2014, regimens for the treatment of advanced melanoma mainly comprised cytotoxic agents such as dacarbazine (DTIC) and cytokines (e.g., type I interferon (IFN), high-dose interleukin (IL)-2, etc.) [1,2,3,4,5,6]. Since 2014, anti-programmed cell death 1 (PD1) antibodies (Abs) have become recognized as anchor drugs for the treatment of advanced melanoma, with or without additional combination drug such as ipilimumab [7,8]. In addition, v-Raf murine sarcoma viral oncogene homolog B1 (BRAF) inhibitors in combination with mitogen-activated protein kinase kinase (MEK) inhibitors are among the most promising chemotherapeutic regimens for the treatment of advanced *BRAF*-mutant melanoma, especially among patients with a low tumor burden [9,10]. Since anti-PD1 Abs are widely applicable to the treatment of both *BRAF* wild-type and mutated advanced melanoma, several clinical trials for drugs combined with anti-PD1 Abs are ongoing—see Section 5. This review focuses on the development of the currently available anti-melanoma therapies, and discusses the clinical trials that might reveal regimens for the future treatment of advanced melanoma.

## 2. IFNs, Cytotoxic Drugs and High-Dose IL-2 for Advanced Melanoma

Before immune checkpoint inhibitors (ICIs), BRAF inhibitors and MEK inhibitors became available in the real world, DTIC, type I IFN and/or IL-2-based combined therapies were the main protocol for the treatment of advanced melanoma [1,2,4,5,6,11,12,13,14,15,16], although the efficacy of those protocols remained insufficient. Among those, the anti-melanoma effects of type I IFN are still controversial. Although the therapeutic effects of type I IFN monotherapy (IFN-α, pegylated IFN-α, IFN-β, etc.) are limited for the treatment of advanced melanoma [4], many protocol regimens that contain Type I IFN have been investigated [1,2,17,18]. For example, Young et al. reported a randomized phase II study comparing the anti-melanoma effects of DTIC plus IFN-α with those of DTIC alone, showing no significant effect of additional IFN-α on overall survival among advanced melanoma patients [1]. In another report, Hauschild et al. undertook a randomized multicenter phase III study comparing the anti-melanoma effects of DTIC plus IFN-α with or without IL-2, finding that response rates did not differ between arms [2]. Grignol et al. reported a phase II study of bevacizumab plus high-dose IFN-α 2b for the treatment of advanced melanoma [19]. They concluded that the clinical response rate among advanced melanoma patients treated with bevacizumab with IFN-alpha was 24%, higher than the historical response rates of 5~13% for IFN-α alone [4,19]. Moreover, Egberts et al. reported a phase II study of sorafenib plus IFN-α 2b for the treatment of metastatic melanoma, showing modest clinical activity (objective response rate (ORR) 3.6%, disease control rate (DCR) 29.1%), but with serious side-effects [5]. In Japan, IFN-β is clinically used for the treatment of advanced melanoma with or without DTIC as an adjuvant therapy [3] or for the treatment of unresectable melanoma [17,20]. Notably, since local injection of IFN-β in melanoma site induces activated PD1-expressing effecter T cells by re-polarizing tumor-associated macrophages (TAMs) [20,21], IFN-β might enhance the anti-melanoma effects of anti-PD1 Abs in patients with unresectable melanoma [17]. Notably, although IFN-α could also re-polarize M2-polarized TAMs into activated M1-like TAMs in the lesional skin of cutaneous T-cell lymphoma [22], a phase Ib clinical trial (KEYNOTE-029) revealed that pegylated IFN-α-2b might not augment the anti-melanoma effects of anti-PD1 Abs in melanoma patients [18]. Those reports suggested that type I IFN might modulate the tumor microenvironment of melanoma, leading to enhanced therapeutic effects from appropriate anti-melanoma drugs, such as anti-PD1 Abs, although the precise mechanisms remain unknown.

DTIC was one of the standard therapies for the treatment of advanced melanoma before ICIs or BRAF inhibitors became available for clinical use [23,24,25,26,27]. Indeed, DTIC was used as a control drug for clinical trials estimating the efficacy of nivolumab [23,24], vemurafenib [25] or dabrafenib [27]. Since the efficacy of DTIC monotherapy is inadequate, several clinical trials have been performed to assess the additional effects of anti-melanoma drugs with DTIC. For example, the anti-melanoma effects of ipilimumab was first estimated as an additional benefit of ipilimumab for DTIC monotherapy [25]. As we described above, the additional benefit of DTIC was evaluated, but no significant effect of additional IFN-α on overall survival was seen for advanced melanoma patients [1]. Daponte et al. reported a phase III study to estimate the therapeutic effects of fotemustine or IFN-α2b with DTIC, although no significant improvement in outcome was achieved [11]. On the other hand, Kaufmann et al. reported a phase III study of temozolomide (the pro-drug of DTIC) plus IFN-α, suggesting that IFN-α significantly enhanced the therapeutic effects of temozolomide [12]. Indeed, the ORR of temozolomide plus IFN-α was 24.1%, compared to 13.4% for temozolomide alone, although hematologic toxicities were significantly more frequent in the temozolomide-plus-IFN-α arm [12]. In Japan, DAVtherapy (DTIC in combination with nimustine hydrochloride (ACNU) and vincristine (VCR)) has been used in the adjuvant setting for advanced melanoma for the last 30 years, although no clinical trial was conducted for DAV therapy with or without IFN-β [13,14]. Notably, and more recently, Fujimura et al. reported immunomodulatory effects of DTIC, ACNU and VCR on TAMs (PD-L1 expression, modulation of Th1/Th2 chemokine production, etc.), suggesting possible immunological mechanisms for DTIC-based anti-melanoma therapy [28].

High-dose interleukin-2 (HD IL-2) therapy has for decades served as one of the immunotherapies for advanced melanoma treatment [6]. Indeed, several reports have suggested that HD IL-2 therapy provides a high ORR for advanced melanoma, particularly when administered as combination therapy [6,15,16,29,30,31]. For example, Keilholz et al. analyzed 631 cases of IL-2-based treatment of stage IV melanoma to compare median survival between patients who treated with IL-2 alone, IL-2 plus chemotherapy, IL-2 plus IFN-alpha and IL-2, and chemotherapy plus IFN-alpha, suggesting that only the addition of IFN-alpha to IL-2 was associated with prolonged survival [6]. Du Bois et al. reported the ORR of HD IL-2-treated advanced melanoma as 22%, augmented by the additional administration of recombinant human soluble p75 tumor necrosis factor receptor immunoglobulin G chimera [15]. Schwartzentruber et al. reported a phase III clinical trial evaluating the additional therapeutic effects of gp100 peptide vaccine on HD IL-2 therapy for advanced melanoma with Human Leukocyte Antigen (HLA)*A0201, suggesting that the vaccine-IL-2 group achieved significant improvements in ORR and progress-free survival (PFS) compared to the HD IL-2-alone group [16]. More recently, Curti et al. reported that stereotactic body radiation therapy enhanced the therapeutic effects of HD IL-2 in the treatment of metastatic melanoma (ORR: 35% vs. 54%), although no differences in PFS or overall survival (OS) were seen [29]. Mooradian et al. reported that while HD IL-2 might increase the ORR to vemurafenib, there was no effect on PFS in patients with *BRAF*V^600E^-mutated advanced melanoma [30]. Since the anti-melanoma response of HD IL-2 differs between genetic subsets of advanced melanoma [31], these HD IL-2-based combination therapies described above might be improved by comprehensive analysis for the driver genes of melanoma before the administration of therapy.

## 3. Clinical Use of BRAF Kinase Inhibitors in the Treatment of *BRAF*-Mutated Advanced Melanoma

### 3.1. Efficacy of BRAF/MEK Inhibitors

The ORRs to BRAF/MEK inhibitor combination therapies for the *BRAF* mutated advanced melanoma are higher than those to other anti-melanoma drugs, including immune checkpoint inhibitors (ICIs) [9,32,33], but the 5-year OS rate and PFS rate with BRAF/MEK inhibitor combination therapies are lower than those with ICIs [7,34]. As a result, BRAF/MEK inhibitor combination therapy is recommended for *BRAF*-mutated advanced melanoma, where early response is clinically needed [35].

Dabrafenib plus trametinib (D + T) is widely used today for the treatment of *BRAF*V^600^-mutated melanoma among the BRAF/MEK inhibitor combination therapies. Pooled analysis of COMBI-d (NCT01584648) and COMBI-v (NCT01597908) demonstrated improved PFS and OS with D + T combination therapy compared with dabrafenib monotherapy [9,10,34]. The ORR to D + T combination therapy is 68%, with complete response (CR) in 19% [34]. Median PFS was 11.1 months (95% confidence interval (CI) 9.5–12.8 months) in the D + T combination therapy group [34]. The 5-year PFS rate for D + T combination therapy in the analysis of that landmark study was 19% (95% CI 15–22%) [34] (Table 1). Notably, patients with a normal baseline lactate dehydrogenase (LDH) level at or below the upper limit of the normal range achieved a 5-year PFS rate of 25% (95% CI 20–30%), as compared with 8% (95% CI 4–13%) in patients with an elevated LDH at baseline [34]. Moreover, multivariate analysis identified that an association was evident between PFS and tumor burden performance status (PS), LDH level and disease site [34]. Median OS was 25.9 months (95% CI 22.6–31.5 months). The 5-year OS rate was 34% (95% CI 30–38%) overall, and was higher among patients with normal LDH at baseline (43% (95% CI 38–49%)) than among those with elevated levels (16% (95% CI 11–22%)). Notably, the estimated 5-year OS rate was 55% (95% CI 48–61%) in patients with normal LDH levels and fewer than three organ sites showing metastases at baseline [34]. Taken together, these data suggested that D + T combined therapy should be selected for patients with *BRAF*V^600^-mutated advanced melanoma who possess a low tumor burden.

Encorafenib plus binimetinib (E + B) combination therapy represents another set of BRAF/MEK inhibitors, and one of the optimal chemotherapies for *BRAF*-mutated advanced melanoma [32,36]. The ORR to E + B combination therapy (as calculated by a masked independent central review) was 64% [37]. E + B combination therapy significantly improved PFS in the E + B combination therapy group compared with an encorafenib monotherapy group (hazard ratio (HR) 0.77, 95% CI 0.59–1.00; *p* = 0.05) [32]. Moreover, the median OS for E + B vs. encorafenib vs. vemurafenib was 33.6 months (95% CI 24.4–39.2 months) vs. 23.5 months (95% CI 19.6–33.6 months) vs. 16.9 months (95% CI 14.0–24.5 months), respectively [32]. Notably, unlike D + T combination therapy, although OS was significantly improved with both E + B combination therapy (E + B vs. vemurafenib: HR 0.61, 95% CI 0.47–0.79; *p* < 0.0001) and encorafenib monotherapy (encorafenib vs. vemurafenib: HR 0.76, 95% CI 0.58–0.98; *p* < 0.033) compared with vemurafenib, no significant difference was evident between E + B combination therapy and encorafenib monotherapy (encorafenib plus binimetinib vs. encorafenib: HR 0.81, 95% CI 0.61–1.06; *p* = 0.12) (Table 1). In aggregate, the analysis in the COLUMBUS (NCT01909453) trial demonstrated improved PFS and OS with E + B combination therapy and encorafenib monotherapy compared with vemurafenib monotherapy [32].

Vemurafenib plus cobimetinib (V + C) combination therapy also improved PFS compared with vemurafenib monotherapy [33]. The analysis of the coBRIM (NCT01689519) trial demonstrated that median PFS for V + C combination therapy was 12.3 months (95% CI 9.5–13.4 months), compared to 7.2 months (95% CI 5.6–7.5 months) for vemurafenib monotherapy (HR 0.58, 95% CI 0.46–0.72; *p* < 0.0001) [33]. Median OS was 22.3 months (95% CI 20.3 months—not estimable) for V + C combination therapy, compared to 17.4 months (95% CI 15.0–19.8 months) for vemurafenib monotherapy (HR 0.70, 95% CI 0.55–0.90; *p* = 0.005). The ORR to V + C combination therapy was 70% (95% CI 63.5–75.3%) [33], which is comparable to the responses seen to D + T combination therapy [34] and E + B combination therapy [32] (Table 1).

### 3.2. Adverse Events with BRAF/MEK Inhibitors

As described above, to date, three different BRAF/MEK inhibitor combinations are available for the treatment of advanced melanoma. All BRAF inhibitors are currently used in combination therapy with MEK inhibitors that can not only improve the antitumor effects (e.g., response rate or duration of response), but also change the profiles of adverse events [9,10,32,33,34,36,40]. Indeed, the incidence of severe pyrexia, chills, diarrhea and vomiting was higher with D + T combination therapy than with dabrafenib monotherapy [9,32]. For example, the incidence ratio of pyrexia was 51% with D + T combined therapy, but 28% with dabrafenib monotherapy [9]. In addition, the incidence ratio of pyrexia was 51% with D + T combined therapy, much higher than with either E + B combination therapy (20%) [32] or V + C combination therapy (29%) [33]. On the other hand, the incidence rate of cutaneous squamous cell carcinoma was lower in the D + T group (2%) than in the dabrafenib group (9%) [9]. The incidence of serous retinopathy was higher with E + B combination therapy (20%) than with encorafenib monotherapy (2%) or vemurafenib monotherapy (2%) [36]. In V + C combination therapy, the incident ratio of serious adverse events was higher in the V + C group (37%) than in a vemurafenib group (28%) [33]. Those reports suggest that the profiles of adverse events (AEs) differ between combination and monotherapy groups for each BRAF/MEK inhibitor pair [9,33,36].

Moreover, subtypes of AEs differed between these three different BRAF/MEK inhibitor pairs [40], which might be caused by the different immunomodulatory effects in melanoma patients [41]. Indeed, a pre-clinical study in a xenografted mouse melanoma model suggested that dabrafenib, but not vemurafenib, enhances the therapeutic effects of anti-PD1 Abs [41]. The investigators concluded that anti-PD1 Abs in combination with D + T therapy might be an optimal protocol for patients with *BRAF*-mutated advanced melanoma. Since the available data from clinical studies mainly comprise results from first-line cohorts, further studies investigating the incidence of AEs in the real world are needed to confirm this hypothesis.

To date, little is known about the mechanisms of AEs developing from melanoma patients treated with BRAF/MEK inhibitors [40,42,43]. Among them, recently, Amagai et al. reported a case series with pyrexia developing from advanced melanoma treated with E + B therapy, and suggested that serum levels of soluble CD163 as well as IFN-γ induced chemokines [C-X-C motif chemokine (CXCL9, CXCL10, CXCL11)] were increased in the pyrexia group compared with the non-pyrexia group [42]. Notably, all of these soluble factors had previously been reported as biomarkers for adult-onset Still’s disease (AOSD) [44,45], and the manifestations of AOSD (including pyrexia, transient skin rash, fatigue and arthritis [44,45]) are well-known AEs developing from BRAF/MEK inhibitors [40]. In another report, Irimada et al. found a correlation between serum CXCL5 levels and onset of rhabdomyolysis caused by D + T therapy [43]. Since all of these soluble factors described above are well-known TAM-related factors [46], BRAF/MEK inhibitors might activate TAMs in advanced melanoma patients directly or indirectly, leading to production of these soluble factors and development of characteristic AEs.

Recent reports have also suggested that BRAF/MEK inhibitors followed by ICIs, or ICIs followed by BRAF/MEK inhibitors, could enhance the incidence of unexpected severe (S)AEs in the real world [37]. For example, two cases of severe Vogt–Koyanagi–Harada (VKH) disease-like uveitis caused by nivolumab followed by D + T combined therapy were reported [47]. Notably, since the patients in both cases possessed a characteristic HLA type that had previously been reported to correlate with the onset of VKH disease [47], the sequential therapy might have triggered the onset of VKH-like AEs. Moreover, two independent case reports about severe rhabdomyolysis caused by ICIs followed by D + T combination therapy for advanced melanoma were reported [43,48]. In addition, nivolumab followed by D + T combination therapy can cause severe drug eruption such as exudative erythema multiforme [49]. Those reports suggest that the subsets and incident ratio of severe adverse events (SAEs) caused by BRAF/MEK inhibitors might differ from those in previously published clinical studies in the real world.

## 4. Clinical Use of ICIs in the Treatment of Advanced Melanoma

### 4.1. Efficacy of Anti-PD1 Antibody Monotherapy against Advanced Melanoma

To date, two different anti-PD1 Abs (nivolumab and pembrolozumab) are available for advanced melanoma treatment [7,8]. Since estimated 5-year OS and 5-year PFS of nivolumab monotherapy for *BRAF*-wild-type advanced melanoma were comparable to those of nivolumab plus ipilimumab (N + I) combination therapy [7], nivolumab monotherapy was considered as a first-line immunotherapy for *BRAF*-wild-type advanced cutaneous melanoma [35]. Five-year OS rate was 44% and 5-year PFS rate was 29% in the nivolumab monotherapy group [7] (Table 1). The ORR of nivolumab monotherapy was 43.7% (95% CI 38.1–49.3%) in a Caucasian population [8], but lower in a Japanese population (34.8%; 95% CI 20.8–51.9%) [50]. The ORR of nivolumab monotherapy in a Japanese population was again much lower than that in a Caucasian population according to post-marketing surveillance analysis in Japan (22.2%) [51]. Notably, the most common subtype of melanoma in Japan was acral lentiginous melanoma (ALM) (40.4%) [52], while the ratio of ALM in a non-Hispanic, white-skinned population in the United States was much lower (1%) [53]. A previous report also suggested that the ORR of anti-PD1 Abs for ALM and mucosal melanoma was lower in a Caucasian population [54]. Taken together, since the ORR of anti-PD1 Abs for advanced ALM in Japanese population was 16.6%, and the PFS and OS for the study cohort were 3.5 months and 18.2 months, respectively [55], the efficacy of anti-PD1 Abs monotherapy for advanced melanoma in the Japanese population was lower than that in the Caucasian population.

Monotherapy with pembrolizumab, another anti-PD1 Ab for advanced melanoma, should also be considered as a first-line immunotherapy for advanced cutaneous melanoma [38,56]. The ORR of pembrolizumab was 37% in melanoma patients taking pembrolizumab every 2 weeks and 36% in melanoma patients taking pembrolizumab every 3 weeks [38]. In a Japanese population, the ORR was 24.1% (95% CI 10.3–43.5%) for cutaneous melanoma and 25.0% (95% CI 3.2–65.1%) for mucosal melanoma [57]. Median OS and median PFS were 32.7 months (95% CI 24.5–41.6 months) and 8.4 months (95% CI 6.6–11.3 months), respectively [56]. The 5-year OS rate was 38.7% (95% CI 34.2–43.1%), and the 48-month PFS rate was 23.0% (95% CI 19.1–27.1%) in the pembrolizumab group [56], suggesting that both 5-year OS and 4-year PFS were comparable to those of nivolumab monotherapy.

Since the efficacies of N + I combination therapy or ipilimumab monotherapy as a second-line immunotherapy are low [58,59], determining the efficacy of nivolumab monotherapy before tumor progression is important. Moreover, the incidence of SAE is much higher in N + I combination therapy groups compared to nivolumab monotherapy groups. For these reasons, biomarkers for predicting the efficacy of nivolumab monotherapy have been under investigation [60]. For example, serum LDH levels should be taken into accounts before selecting immunotherapy [7,61]. Indeed, subgroup analysis from the CheckMate 067 (NCT01844505) trial confirmed a correlation of efficacy of nivolumab monotherapy with serum LDH levels, and suggested that nivolumab monotherapy should also be considered as a first-line immunotherapy for advanced cutaneous melanoma in patients with normal LDH levels [7]. In addition, a pre-clinical study suggested that an increased baseline neutrophil-lymphocyte ratio combined with normal serum LDH correlated significantly with the efficacy rate of nivolumab according to multivariate analysis [61]. These reports suggested the significance of serum LDH to predict the efficacy of nivolumab monotherapy. In addition, subgroup analysis of the CheckMate 067 (NCT01844505) trial also suggested that tumor PD-L1 expression alone was not predictive of efficacy outcomes [7], although other previous reports suggested that PD-L1 expression on melanoma cells can represent a biomarker for predicting the efficacy of anti-PD1 Abs [60,62,63].

More recently, TAM-related factors (soluble (s)CD163, CXCL5, (Chemokine (C-C motif) ligand (CCL)19 and CCL26)) could be predictive biomarkers for anti-PD1 Abs monotherapy [64,65,66,67]. In future, these predictive markers might be taken into accounts in combination with conventional markers, such as *BRAF* mutation, before selecting the protocol for immune therapy.

### 4.2. Efficacy of N + I Combination Therapy against Advanced Melanoma

N + I combination therapy is currently the most effective therapy for advanced melanoma [7,8], and is recommended as a first-line immunotherapy for the treatment of advanced melanoma [35]. The ORR to N + I combination therapy was higher than that to nivolumab monotherapy (57.6% (95% CI 52.0–63.2%) vs. 43.7% (95% CI 38.1–49.3%)) [8] (Table 1). The percentage of patients showing complete response was higher in the N + I combination group (11.5%) than in the nivolumab monotherapy group (8.9%) [8]. Notably, among patients with elevated LDH levels and high tumor burden, 5-year OS rate was much better with N + I combination therapy (38%) than with nivolumab monotherapy (28%). Five-year PFS rate was also improved with N + I combination therapy (28%) compared to nivolumab monotherapy (18%) among patients with elevated LDH levels and high tumor burden [7]. Indeed, a recent case report described a patient with advanced melanoma and 7 metastatic organs successfully treated using N + I combination therapy in the real world [39] Subgroup analysis of the CheckMate 067 trial revealed the utility of N + I combination therapy for the *BRAF*-mutated advanced melanoma group [7]. Indeed, the 5-year OS rate was much improved in the N + I combination group (60%) than in the nivolumab monotherapy group (46%) among patients with tumors with *BRAF* mutations [7]. Five-year PFS rate was also improved with N + I combination therapy (38%) compared to with nivolumab monotherapy (22%) among patients with tumors showing *BRAF* mutations. Notably, these 5-year OS and PFS rates were comparable to those of D + T combination therapy [34]. Since the efficacy of BRAF/MEK inhibitors among patients with elevated LDH levels is limited [34], N + I combination therapy could be a first-line immunotherapy for *BRAF*-mutated advanced melanoma with high tumor burden.

### 4.3. Other ICIs Related Protocol

Recent NCCN Clinical Practice Guidelines for cutaneous melanoma also recommended anti-PD-L1 antibodies in combination with BRAF/MEK inhibitor [36]. Gutzmer et al. reported the efficacy of atezolizumab plus V + C (A + V + C) combination therapy for unresectable *BRAF^V600^* mutation-positive melanoma [68]. Although ORR of A + V + C combination therapy (66.3% (95% CI; 60.1–72.1)) was similar to V + C combination therapy (65.0% (95% CI; 58.7–72.1)), median PFS favored in A + V + C combination group (16.1 months (95%; CI 11.3–18.5)) compared with V + C combination therapy (12.3 months (95% CI; 10.8–14.7) (HR 0.85; 95% CI 0.67–1.07; log-rank *p* = 0.16)).

Talimogene Laherparepvec (T-vec) should also be taken into accounts for the treatment of advanced melanoma in combination with or without ICIs [69,70]. As Chesney et al. reported, the ORR of T-vec plus ipilimumab (39%) was higher than that of ipilimumab (18%) (odds ratio: 2.9 (95% CI; 1.5 to 5.5; *p* = 0.002)) in patients with advanced melanoma with prior therapy [70]. In another report, T-vec monotherapy was evaluated [70]. For patients with stage IV-M1a melanoma, the effect of T-VEC on DRR was higher than that of control cohort (recombinant GM-CSF) (24.0% vs. 0%). For patients with stage IV-M1b or -M1c disease, however, the effects of T-VEC on DRR and OS were small and not statistically significant [70].

### 4.4. Immune-Related AEs (irAEs)

As described above, anti-PD-1 Abs significantly prolong survival in patients with metastatic melanoma, and co-administration with ipilimumab leads to further improved outcomes [7,8]. However, co-administration of nivolumab and ipilimumab and sequential administration of nivolumab and ipilimumab with a planned switch leads to a high frequency of irAEs among patients with advanced melanoma [7,8,71]. Indeed, safety profiles from the CheckMate 067 trial revealed that the incidence of treatment-related SAE was higher with N + I combination therapy (55.0%) than with nivolumab monotherapy (16.3%) [8]. Among these, the incidence of treatment-related colitis, hepatitis and skin rash was 5 to 10 times much higher with N + I combination therapy than with nivolumab monotherapy [8]. Moreover, several reports have suggested that AEs caused by ICIs correlate with better response among melanoma patients [64,72,73,74]. For example, Kobayashi et al. reported that melanoma patients who developed pituitary dysfunction induced by ICIs display better OS than patients without this irAE [72]. Schadendorf et al. reported that the ORR to N + I combination therapy was higher in patients who discontinued because of irAEs during the induction phase than in patients who did not discontinue [73]. Since absolute serum levels of sCD163 were significantly increased in patients who developed AEs [74], and serum levels of sCD163 were also significantly increased in melanoma patients who responded to nivolumab monotherapy [64], irAEs caused by ICIs might correlate with the efficacy of nivolumab. Indeed, serum levels of sCD163 in a patient with isolated ACTH deficiency caused by nivolumab were increased with good response to nivolumab monotherapy [72]. These reports suggest that several subtypes of irAEs caused by ICIs might correlate with better response in melanoma patients.

The correlation between human leukocyte antigen (HLA) subtypes and several irAEs caused by ICIs has been investigated in various studies [47,75,76,77,78,79]. For example, Fujimura et al. reported two cases of advanced melanoma patients who possessed HLA-DRB1*04:05, which is strongly associated with VKH disease, developing VKH-like uveitis after sequential administration of nivolumab and D + T combination therapy [47]. In other report, Magis et al. reported that in acute type I diabetes, 80% of patients (4/5) possessed HLA-DRB01*03 or *04 subtypes, which are known to increase type 1 diabetes risk in the general population [73]. Kambayashi et al. also reported a case of severe demyelinating neuropathy in an advanced melanoma patient treated with N + I combination therapy, who showed HLA-DQB1 polymorphisms (DQB1*040101 and *060401) [77]. More recently, Yano et al. reported that HLA-DR15, which is reported to correlate with autoimmune disease via IL-17 regulation, could be a predictive marker for ICI-induced secondary adrenal insufficiency [78]. Since both IL-17 and sCD163 reportedly correlate with autoimmune skin diseases such as bullous pemphigoid [79], psoriasis [80] and alopecia areata [81], and these autoimmune skin diseases were occasionally induced by ICIs in melanoma patients [82,83,84], HLA genotyping before treatment may help to avoid the incidence of irAEs by ICIs.

## 5. Future Perspectives

Since both BRAF/MEK inhibitors and ICIs could obtain the resistance [85,86,87,88], several pre-clinical studies had performed before the clinical studies on going today. For example, Corre et al. reported that aryl hydrocarbon receptor (AhR) transcription factor constitutively activates human melanoma cells to induce BRAF inhibitor-resistance genes [89]. In addition, targeting AhR signaling could prevent the induction of the BRAF inhibitor resistance gene in melanoma cells [87]. Another report suggested that resistance to BRAF/MEK inhibitors combined therapy is associated with reactive oxygen species (ROS) activities in human melanoma cell lines, and the administration of a histone deacetylase (HDAC) inhibitor induces selective apoptotic death of drug-resistant tumor cells [85]. Since short-term treatment with the HDAC inhibitor can eliminate cells harboring secondary mutations that induce resistance of BRAF and MEK inhibitors [86], HDAC inhibitors are a possible combined drug for BRAF/MEK inhibitors. Indeed, a phase III trial to evaluate sequential treatment of vorinostat and BRAF inhibitors plus MEK inhibitors in resistant BRAFV^600E^ mutant melanoma is ongoing (NCT02836548).

As described above, ICIs are currently among the most promising methods for inducing long-acting anti-tumor effects [7]. Notably, a recent pre-clinical study suggested possible novel therapies for the treatment of advanced melanoma using therapy combining BRAF/MEK inhibitors and ICIs [41]. Indeed, several phase I/II clinical studies for the treatment of patients with advanced melanoma have been set up according to such preclinical studies (Table 2). For example, a phase I/II study to assess the safety and efficacy of pembrolizumab in combination with dabrafenib and trametinib for *BRAF*-mutated advanced melanoma is now ongoing (NCT02130466). Since a dose-seeking and efficacy study of pembrolizumab plus vemurafenib and cobimetinib for advanced melanoma is also ongoing (NCT02818023), results from these two independent clinical trials should be compared. In addition, a phase II clinical trial to evaluate sequential administration of BRAF/MEK inhibitors, V + C combination therapy and N + I combination therapy for the treatment of *BRAF*-mutated advanced melanoma is ongoing (NCT02968303). The results of such clinical trials might offer more optimal protocols for *BRAF*-mutated advanced melanoma in the future. Unlike anti-PD1 Abs, ipilimumab is unsuitable for sequential therapy with BRAF inhibitors because of the high frequency of severe hepatotoxicity [85].

Several clinical trials for *BRAF*-wild-type advanced melanoma using anti-PD1 Ab-based immunotherapy are ongoing. Among these, Rozeman et al. reported a phase 2 clinical study of N + I combination therapy in a neoadjuvant setting for macroscopic stage III melanoma (OpACIN-neo) [89]. That clinical trial suggested appropriate doses of 1 mg/kg for ipilimumab and 3 mg/kg for nivolumab in a neoadjuvant setting to induce pathological response in a high proportion of patients without SAEs [90]. Indeed, in this dose setting, the ORR was 57% (17/30) and no grade 3–4 AEs were seen in more than one patient each, suggesting that this protocol might be suitable for broader clinical use against stage III melanoma involving lymph nodes [90].

Since a pre-clinical report has suggested that blockade of the receptor of receptor activator of nuclear factor kappa-B (RANK) enhanced the therapeutic effects of ICIs in a B16F10 melanoma model [89], denosumab might be suitable for treating advanced melanoma [91]. Indeed, based on such pre-clinical studies, a phase II study to assess the efficacy of anti-PD1 Abs (nivolumab or pembrolizumab) in combination with denosumab for stage III/IV melanoma is now underway (NCT03620019). In other pre-clinical studies, IFN-β also enhanced the therapeutic effects of anti-PD1 Abs in a B16F10 melanoma model by recruiting cytotoxic CD8+ T cells to tumor sites [20,21].

According to those pre-clinical studies, a phase 1 clinical trial had already been performed [17]. According to that clinical trial, the efficacy of anti-PD1 Abs might be improved without severe irAEs in future [17,85].

## Figures and Tables

**Table 1:** Firstline therapy recommended by National Comprehensive Cancer Network (NCCN) guideline 2020.4.

Protocol	Efficacy	Median OS (95% CI)	5-Year OS	Median PFS (95% CI)	5-Year PFS	Reference
nivolumab monotherapy	43.7%	36.9 M	44.0%	11.5 M	29.0%	[7]
pembtolizumab monotherapy	36.0%	32.7 M	38.7%	8.4 M	23.0% (4-year)	[38]
N + I combination therapy	57.6%	60 M	52.0%	6.9 M	36.0%	[7]
D + T combination therapy	68.0%	25.9 M (22.6–31.5)	34.0%	11.1 M (9.5–12.8)	19.0%	[34]
V + C combination therapy	70.0%	22.3 months (20·3–N.E.)		12.3 M (9.5–13.4)		[33]
E + B combination therapy	64.0%	33.6 M (24.4–39.2)		14.9 M (11.0–20.2)		[32]
A + V + C combination therapy	66.3%	N.E. (2 years)		16.1 M (11.3–18.5)		[39]

N.E.: not estimable; N + I: nivolumab + ipilimumab; D + T: dabrafenib + trametinib; V + C: vemurafenib + cobimetinib; E + B: encorafenib + binimetinib; A + V + C: atezolizumab + V + C.

**Table 2:** Clinical trials on going.

Phase	Protocol	Cancer Species	Reference
I/II	pembrolizumab plus D + T	*BRAF*-mutated advanced melanoma	NCT02130466
I	pembrolizumab plus V + C	*BRAF*-mutated advanced melanoma	NCT02818023
II	sequential V + C and N + I	*BRAF*-mutated advanced melanoma	NCT02968303
II	neoadjuvant N + I	macroscopic stage III melanoma	[89]
II	anti-PD1 Abs plus denosumab	stage III/IV melanoma	NCT03620019
III	vorinostat plus BRAFi/MEKi	resistant *BRAFV*^600E^ mutant melanoma	NCT02836548

## References

[1] Young A.M., Marsden J., Goodman A., Burton A., Dunn J.A. (2001). Prospective randomized comparison of dacarbazine (DTIC) versus DTIC plus interferon-alpha (IFN-alpha) in metastatic melanoma. Clin. Oncol..

[2] Hauschild A., Garbe C., Stolz W., Ellwanger U., Seiter S., Dummer R., Ugurel S., Sebastian G., Nashan D., Linse R. (2001). Dacarbazine and interferon alpha with or without interleukin 2 in metastatic melanoma: A randomized phase III multicentre trial of the Dermatologic Cooperative Oncology Group (DeCOG). Br. J. Cancer.

[3] Namikawa K., Tsutsumida A., Mizutani T., Shibata T., Takenouchi T., Yoshikawa S., Kiyohara Y., Uchi H., Furue M., Ogata D. (2017). Randomized phase III trial of adjuvant therapy with locoregional interferon beta versus surgery alone in stage II/III cutaneous melanoma: Japan Clinical Oncology Group Study (JCOG1309, J-FERON). Jpn. J. Clin. Oncol..

[4] Legha S.S. (1997). The role of interferon alfa in the treatment of metastatic melanoma. Semin. Oncol..

[5] Egberts F., Gutzmer R., Ugurel S., Becker J.C., Trefzer U., Degen A., Schenck F., Frey L., Wilhelm T., Hassel J.C. (2011). Sorafenib and pegylated interferon-α2b in advanced metastatic melanoma: A multicenter phase II DeCOG trial. Ann. Oncol..

[6] Keilholz U., Conradt C., Legha S.S., Khayat D., Scheibenbogen C., Thatcher N., Goey S.H., Gore M., Dorval T., Hancock B. (1998). Results of interleukin-2-based treatment in advanced melanoma: A case record-based analysis of 631 patients. J. Clin. Oncol..

[7] Larkin J., Chiarion-Sileni V., Gonzalez R., Grob J.J., Rutkowski P., Lao C.D., Cowey C.L., Schadendorf D., Wagstaff J., Dummer R. (2019). Five-Year Survival with Combined Nivolumab and Ipilimumab in Advanced Melanoma. N. Engl. J. Med..

[8] Larkin J., Chiarion-Sileni V., Gonzalez R., Grob J.J., Cowey C.L., Lao C.D., Schadendorf D., Dummer R., Smylie M., Rutkowski P. (2015). Combined Nivolumab and Ipilimumab or Monotherapy in Untreated Melanoma. N. Engl. J. Med..

[9] Long G.V., Stroyakovskiy D., Gogas H., Levchenko E., de Braud F., Larkin J., Garbe C., Jouary T., Hauschild A., Grob J.J. (2014). Combined BRAF and MEK inhibition versus BRAF inhibition alone in melanoma. N. Engl. J. Med..

[10] Long G.V., Flaherty K.T., Stroyakovskiy D., Gogas H., Levchenko E., de Braud F., Larkin J., Garbe C., Jouary T., Hauschild A. (2017). Dabrafenib plus trametinib versus dabrafenib monotherapy in patients with metastatic *BRAF*^V600E^/K-mutant melanoma: Long-term survival and safety analysis of a phase 3 study. Ann. Oncol..

[11] Daponte A., Signoriello S., Maiorino L., Massidda B., Simeone E., Grimaldi A.M., Caracò C., Palmieri G., Cossu A., Botti G. (2013). Southern Italy Cooperative Oncology Group (SICOG). Phase III randomized study of fotemwustine and dacarbazine versus dacarbazine with or without interferon-α in advanced malignant melanoma. J. Transl. Med..

[12] Kaufmann R., Spieth K., Leiter U., Mauch C., von den Driesch P., Vogt T., Linse R., Tilgen W., Schadendorf D., Becker J.C. (2005). Dermatologic Cooperative Oncology Group. Temozolomide in combination with interferon-alfa versus temozolomide alone in patients with advanced metastatic melanoma: A randomized, phase III, multicenter study from the Dermatologic Cooperative Oncology Group. J. Clin. Oncol..

[13] Matsumoto T., Yokota K., Sawada M., Sakakibara A., Shibata S., Yasue S., Tomita Y., Yatsuya H., Akiyama M. (2013). Postoperative DAV-IFN-β therapy does not improve survival rates of stage II and stage III melanoma patients significantly. J. Eur. Acad. Dermatol. Venereol..

[14] Umeda T., Aoki K., Yokoyama A., Ohara H., Hayashi O., Tanaka K., Nishioka K. (1998). Changes in immunological parameters after combination adjuvant therapy with intravenous DTIC, ACNU, and VCR, and local injection of IFN-beta (DAV + IFN-beta therapy) into malignant melanoma. J. Dermatol..

[15] Du Bois J.S., Trehu E.G., Mier J.W., Shapiro L., Epstein M., Klempner M., Dinarello C., Kappler K., Ronayne L., Rand W. (1997). Randomized placebo-controlled clinical trial of high-dose interleukin-2 in combination with a soluble p75 tumor necrosis factor receptor immunoglobulin G chimera in patients with advanced melanoma and renal cell carcinoma. J. Clin. Oncol..

[16] Schwartzentruber D.J., Lawson D.H., Richards J.M., Conry R.M., Miller D.M., Treisman J., Gailani F., Riley L., Conlon K., Pockaj B. (2011). gp100 peptide vaccine and interleukin-2 in patients with advanced melanoma. N. Engl. J. Med..

[17] Fujimura T., Hidaka T., Kambayashi Y., Furudate S., Kakizaki A., Tono H., Tsukada A., Haga T., Hashimoto A., Morimoto R. (2017). Phase I study of nivolumab combined with IFN-β for patients with advanced melanoma. Oncotarget.

[18] Atkins M.B., Hodi F.S., Thompson J.A., McDermott D.F., Hwu W.J., Lawrence D.P., Dawson N.A., Wong D.J., Bhatia S., James M. (2018). Pembrolizumab Plus Pegylated Interferon alfa-2b or Ipilimumab for Advanced Melanoma or Renal Cell Carcinoma: Dose-Finding Results from the Phase Ib KEYNOTE-029 Study. Clin. Cancer Res..

[19] Grignol V.P., Olencki T., Relekar K., Taylor C., Kibler A., Kefauver C., Wei L., Walker M.J., Chen H.X., Kendra K. (2011). A phase 2 trial of bevacizumab and high-dose interferon alpha 2B in metastatic melanoma. J. Immunother..

[20] Fujimura T., Okuyama R., Ohtani T., Ito Y., Haga T., Hashimoto A., Aiba S. (2009). Perilesional treatment of metastatic melanoma with interferon-beta. Clin. Exp. Dermatol..

[21] Kakizaki A., Fujimura T., Furudate S., Kambayashi Y., Yamauchi T., Yagita H., Aiba S. (2015). Immunomodulatory effect of peritumoral administration of interferon-beta on melanoma through tumor-associated macrophages. Oncoimmunology.

[22] Furudate S., Fujimura T., Kakizaki A., Hidaka T., Asano M., Aiba S. (2016). Tumor-associated M2 macrophages in mycosis fungoides acquire immunomodulatory function by interferon alpha and interferon gamma. J. Dermatol. Sci..

[23] Ascierto P.A., Long G.V., Robert C., Brady B., Dutriaux C., Di Giacomo A.M., Mortier L., Hassel J.C., Rutkowski P., McNeil C. (2019). Survival Outcomes in Patients with Previously Untreated *BRAF* Wild-Type Advanced Melanoma Treated with Nivolumab Therapy: Three-Year Follow-up of a Randomized Phase 3 Trial. JAMA Oncol..

[24] Robert C., Long G.V., Brady B., Dutriaux C., Maio M., Mortier L., Hassel J.C., Rutkowski P., McNeil C., Kalinka-Warzocha E. (2015). Nivolumab in previously untreated melanoma without BRAF mutation. N. Engl. J. Med..

[25] Maio M., Grob J.J., Aamdal S., Bondarenko I., Robert C., Thomas L., Garbe C., Chiarion-Sileni V., Testori A., Chen T.T. (2015). Five-year survival rates for treatment-naive patients with advanced melanoma who received ipilimumab plus dacarbazine in a phase III trial. J. Clin. Oncol..

[26] Chapman P.B., Robert C., Larkin J., Haanen J.B., Ribas A., Hogg D., Hamid O., Ascierto P.A., Testori A., Lorigan P.C. (2017). Vemurafenib in patients with *BRAFV*^600^ mutation-positive metastatic melanoma: Final overall survival results of the randomized BRIM-3 study. Ann. Oncol..

[27] Hauschild A., Grob J.J., Demidov L.V., Jouary T., Gutzmer R., Millward M., Rutkowski P., Blank C.U., Miller W.H., Kaempgen E. (2012). Dabrafenib in *BRAF*-mutated metastatic melanoma: A multicentre, open-label, phase 3 randomised controlled trial. Lancet.

[28] Fujimura T., Kakizaki A., Kambayashi Y., Sato Y., Tanita K., Lyu C., Furudate S., Aiba S. (2018). Cytotoxic anti-melanoma drugs suppress the activation of M2 macrophages. Exp. Dermatol..

[29] Curti B., Crittenden M., Seung S.K., Fountain C.B., Payne R., Chang S., Fleser J., Phillips K., Malkasian I., Dobrunick L.B. (2020). Randomized phase II study of stereotactic body radiotherapy and interleukin-2 versus interleukin-2 in patients with metastatic melanoma. J. Immunother. Cancer.

[30] Mooradian M.J., Reuben A., Prieto P.A., Hazar-Rethinam M., Frederick D.T., Nadres B., Piris A., Juneja V., Cooper Z.A., Sharpe A.H. (2018). A phase II study of combined therapy with a BRAF inhibitor (vemurafenib) and interleukin-2 (aldesleukin) in patients with metastatic melanoma. Oncoimmunology.

[31] Joseph R.W., Sullivan R.J., Harrell R., Stemke-Hale K., Pankam D., Manoukian G., Percy A., Bassett R.L., Ng C.S., Radvanyi L. (2012). Correlation of NRAS mutations with clinical response to high-dose IL-2 in patients with advanced melanoma. J. Immunother..

[32] Gogas H.J., Flaherty K.T., Dummer R., Ascierto P.A., Arance A., Mandala M., Liszkay G., Garbe C., Schadendorf D., Krajsova I. (2018). Overall survival in patients with *BRAF*-mutant melanoma receiving encorafenib plus binimetinib versus vemurafenib or encorafenib (COLUMBUS): A multicentre, open-label, randomised, phase 3 trial. Lancet Oncol..

[33] Ascierto P.A., McArthur G.A., Dréno B., Atkinson V., Liszkay G., Di Giacomo A.M., Mandalà M., Demidov L., Stroyakovskiy D., Thomas L. (2016). Cobimetinib combined with vemurafenib in advanced *BRAF*(V600)-mutant melanoma (coBRIM): Updated efficacy results from a randomised, double-blind, phase 3 trial. Lancet Oncol..

[34] Robert C., Grob J.J., Stroyakovskiy D., Karaszewska B., Hauschild A., Levchenko E., Chiarion Sileni V., Schachter J., Garbe C., Bondarenko I. (2019). Five-Year Outcomes with Dabrafenib plus Trametinib in Metastatic Melanoma. N. Engl. J. Med..

[35] NCCN Clinical Practice Guidelines in Oncology (NCCN Guidelines®) Cutaneous Melanoma Version 4. https://www.nccn.org/professionals/physician_gls/pdf/cutaneous_melanoma.pdf.

[36] Gogas H.J., Flaherty K.T., Dummer R., Ascierto P.A., Arance A., Mandala M., Liszkay G., Garbe C., Schadendorf D., Krajsova I. (2019). Adverse events associated with encorafenib plus binimetinib in the COLUMBUS study: Incidence, course and management. Eur. J. Cancer.

[37] Kroeze S.G., Fritz C., Hoyer M., Lo S.S., Ricardi U., Sahgal A., Stahel R., Stupp R., Guckenberger M. (2017). Toxicity of concurrent stereotactic radiotherapy and targeted therapy or immunotherapy: A systematic review. Cancer Treat. Rev..

[38] Schachter J., Ribas A., Long G.V., Arance A., Grob J.J., Mortier L., Daud A., Carlino M.S., McNeil C., Lotem M. (2017). Pembrolizumab versus ipilimumab for advanced melanoma: Final overall survival results of a multicentre, randomised, open-label phase 3 study (KEYNOTE-006). Lancet.

[39] Gutzmer R., Stroyakovskiy D., Gogas H., Robert C., Lewis K., Protsenko S., Pereira R.P., Eigentler T., Rutkowski P., Demidov L. (2020). Atezolizumab, vemurafenib, and cobimetinib as first-line treatment for unresectable advanced *BRAF*(V600) mutation-positive melanoma (IMspire150): Primary analysis of the randomised, double-blind, placebo-controlled, phase 3 trial. Lancet.

[40] Greco A., Safi D., Swami U., Ginader T., Milhem M., Zakharia Y. (2019). Efficacy and Adverse Events in Metastatic Melanoma Patients Treated with Combination BRAF Plus MEK Inhibitors Versus BRAF Inhibitors: A Systematic Review. Cancers.

[41] Hu-Lieskovan S., Mok S., Homet Moreno B., Tsoi J., Robert L., Goedert L., Pinheiro E.M., Koya R.C., Graeber T.G., Comin-Anduix B. (2015). Improved antitumor activity of immunotherapy with BRAF and MEK inhibitors in *BRAF*(V600E) melanoma. Sci. Transl. Med..

[42] Amagai R., Fujimura T., Kambayashi Y., Sato Y., Tanita K., Ohuchi K., Hashimoto A., Aiba S. (2020). Severe pyrexia from nivolumab-resistant advanced melanoma after successful combined therapy with encorafenib plus binimetinib. J. Dermatol..

[43] Irimada M., Fujimura T., Kambayashi Y., Tsukada A., Takahashi T., Hashimoto A., Aiba S. (2018). Severe rhabdomyolysis developing from an advanced melanoma patient treated by pembrolizumab followed by dabrafenib trametinib combined therapy. J. Dermatol..

[44] Colafrancesco S., Priori R., Alessandri C., Astorri E., Perricone C., Blank M., Agmon-Levin N., Shoenfeld Y., Valesini G. (2014). sCD163 in AOSD: A biomarker for macrophage activation related to hyperferritinemia. Immunol. Res..

[45] Han J.H., Suh C.H., Jung J.Y., Ahn M.H., Han M.H., Kwon J.E., Yim H., Kim H.A. (2017). Elevated circulating levels of the interferon-γ-induced chemokines are associated with disease activity and cutaneous manifestations in adult-onset Still’s disease. Sci. Rep..

[46] Fujimura T., Aiba S. (2020). Significance of immunosuppressive cells as a target for immunotherapies in melanoma and non-melanoma skin cancers. Biomolecules.

[47] Fujimura T., Kambayashi Y., Tanita K., Sato Y., Hidaka T., Otsuka A., Tanaka H., Furudate S., Hashimoto A., Aiba S. (2018). Two cases of Vogt-Koyanagi Harada disease-like uveitis developing from an advanced melanoma patient treated by sequential administration of nivolumab and dabrafenib/trametinib therapy. J. Dermatol..

[48] Muto Y., Ng W., Namikawa K., Takahashi A., Tsutsumida A., Nishida M., Yamazaki N. (2018). Success of rechallenging dabrafenib and trametinib combination therapy after trametinib-induced rhabdomyolysis: A case report. Melanoma Res..

[49] Fujimura T., Kambayashi Y., Hidaka T., Tamabuchi E., Ohtake E., Tono H., Mizuashi M., Furudate S., Aiba S. (2018). Severe erythema exudative multiforme developing from advanced melanoma treated by dabrafenib and trametinib followed by nivolumab. J. Dermatol..

[50] Yamazaki N., Kiyohara Y., Uhara H., Uehara J., Fujimoto M., Takenouchi T., Otsuka M., Uchi H., Ihn H., Minami H. (2017). Efficacy and safety of nivolumab in Japanese patients with previously untreated advanced melanoma: A phase II study. Cancer Sci..

[51] Kiyohara Y., Uhara H., Ito Y., Matsumoto N., Tsuchida T., Yamazaki N. (2018). Safety and efficacy of nivolumab in Japanese patients with malignant melanoma: An interim analysis of a postmarketing surveillance. J. Dermatol..

[52] Fujisawa Y., Yoshikawa S., Minagawa A., Takenouchi T., Yokota K., Uchi H., Noma N., Nakamura Y., Asai J., Kato J. (2019). Clinical and histopathological characteristics and survival analysis of 4594 Japanese patients with melanoma. Cancer Med..

[53] Bradford P.T., Goldstein A.M., McMaster M.L., Tucker M.A. (2009). Acral lentiginous melanoma: Incidence and survival patterns in the United States, 1986–2005. Arch. Dermatol..

[54] Shoushtari A.N., Munhoz R.R., Kuk D., Ott P.A., Johnson D.B., Tsai K.K., Rapisuwon S., Eroglu Z., Sullivan R.J., Luke J.J. (2016). The efficacy of anti-PD-1 agents in acral and mucosal melanoma. Cancer.

[55] Nakamura Y., Namikawa K., Yoshino K., Yoshikawa S., Uchi H., Goto K., Nakamura Y., Fukushima S., Kiniwa Y., Takenouchi T. (2020). Anti-PD1 checkpoint inhibitor therapy in acral melanoma: A multicenter study of 193 Japanese patients. Ann. Oncol..

[56] Robert C., Ribas A., Schachter J., Arance A., Grob J.J., Mortier L., Daud A., Carlino M.S., McNeil C.M., Lotem M. (2019). Pembrolizumab versus ipilimumab in advanced melanoma (KEYNOTE-006): Post-hoc 5-year results from an open-label, multicentre, randomised, controlled, phase 3 study. Lancet Oncol..

[57] Yamazaki N., Takenouchi T., Fujimoto M., Ihn H., Uchi H., Inozume T., Kiyohara Y., Uhara H., Nakagawa K., Furukawa H. (2017). Phase 1b study of pembrolizumab (MK-3475; anti-PD-1 monoclonal antibody) in Japanese patients with advanced melanoma (KEYNOTE-041). Cancer Chemother. Pharmacol..

[58] Fujisawa Y., Yoshino K., Otsuka A., Funakoshi T., Uchi H., Fujimura T., Matsushita S., Hata H., Okuhira H., Tanaka R. (2018). Retrospective study of advanced melanoma patients treated with ipilimumab after nivolumab: Analysis of 60 Japanese patients. J. Dermatol. Sci..

[59] Kreft S., Gesierich A., Eigentler T., Franklin C., Valpione S., Ugurel S., Utikal J., Haferkamp S., Blank C., Larkin J. (2019). Efficacy of PD-1-based immunotherapy after radiologic progression on targeted therapy in stage IV melanoma. Eur. J. Cancer.

[60] Kambayashi Y., Fujimura T., Hidaka T., Aiba S. (2019). Biomarkers for the prediction of efficacies of anti-PD1 antibodies: Mini reviews. Front. Med..

[61] Fujisawa Y., Yoshino K., Otsuka A., Funakoshi T., Fujimura T., Yamamoto Y., Hata H., Tanaka R., Yamaguchi K., Nonomura Y. (2018). Baseline neutrophil to lymphocyte ratio and serum LDH level associated with outcome of nivolumab immunotherapy in Japanese advanced melanoma population. Br. J. Dermatol..

[62] Carlino M.S., Long G.V., Schadendorf D., Robert C., Ribas A., Richtig E., Nyakas M., Caglevic C., Tarhini A., Blank C. (2018). Outcomes by line of therapy and programmed death ligand 1 expression in patients with advanced melanoma treated with pembrolizumab or ipilimumab in KEYNOTE-006: A randomised clinical trial. Eur. J. Cancer.

[63] Daud A.I., Wolchok J.D., Robert C., Hwu W.J., Weber J.S., Ribas A., Hodi F.S., Joshua A.M., Kefford R., Hersey P. (2016). Programmed Death-Ligand 1 Expression and Response to the Anti-Programmed Death 1 Antibody Pembrolizumab in Melanoma. J. Clin. Oncol..

[64] Fujimura T., Sato Y., Tanita K., Kambayashi Y., Otsuka A., Fujisawa Y., Yoshino K., Matsushita S., Funakoshi T., Hata H. (2018). Serum level of soluble CD163 may be a predictive marker of the effectiveness of nivolumab in patients with advanced cutaneous melanoma. Front. Oncol..

[65] Fujimura T., Sato Y., Tanita K., Lyu C., Kambayashi Y., Amagai R., Otsuka A., Fujisawa Y., Yoshino K., Matsushita S. (2019). Association of baseline serum levels of CXCL5 with the efficacy of nivolumab in advanced melanoma. Front. Med..

[66] Fujimura T., Sato Y., Tanita K., Lyu C., Kambayashi Y., Fujisawa Y., Uchi H., Yamamoto Y., Otsuka A., Yoshino K. (2020). Immune checkpoint inhibitor-induced vitiligo in advanced melanoma could be related to increased levels of CCL19. Br. J. Dermatol..

[67] Fujimura T., Tanita K., Ohuchi K., Sato Y., Lyu C., Kambayashi Y., Fujisawa Y., Tanaka R., Hashimoto A., Aiba S. (2020). Increased serum CCL26 level is a potential biomarker for the effectiveness of anti-PD1 antibodies in patients with advanced melanoma. Melanoma Res..

[68] Fujimura T., Kambayashi Y., Sato T., Tanita K., Amagai R., Hashimoto A., Hidaka T., Aiba S. (2019). Successful treatment of unresectable advanced melanoma with pre-surgical administration of nivolumab with ipilimumab. Front. Med..

[69] Chesney J., Puzanov I., Collichio F., Singh P., Milhem M.M., Glaspy J., Hamid O., Ross M., Friedlander P., Garbe C. (2018). Randomized, Open-Label Phase II Study Evaluating the Efficacy and Safety of Talimogene Laherparepvec in Combination with Ipilimumab Versus Ipilimumab Alone in Patients with Advanced, Unresectable Melanoma. J. Clin. Oncol..

[70] Andtbacka R.H.I., Collichio F., Harrington K.J., Middleton M.R., Downey G., Ӧhrling K., Kaufman H.L. (2019). Final analyses of OPTiM: A randomized phase III trial of talimogene laherparepvec versus granulocyte-macrophage colony-stimulating factor in unresectable stage III-IV melanoma. J. Immunother. Cancer.

[71] Weber J.S., Gibney G., Sullivan R.J., Sosman J.A., Slingluff C.L., Lawrence D.P., Logan T.F., Schuchter L.M., Nair S., Fecher L. (2016). Sequential administration of nivolumab and ipilimumab with a planned switch in patients with advanced melanoma (CheckMate 064): An open-label, randomised, phase 2 trial. Lancet Oncol..

[72] Kobayashi T., Iwama S., Yasuda Y., Okada N., Okuji T., Ito M., Onoue T., Goto M., Sugiyama M., Tsunekawa T. (2020). Pituitary dysfunction induced by immune checkpoint inhibitors is associated with better overall survival in both malignant melanoma and non-small cell lung carcinoma: A prospective study. J. Immunother. Cancer.

[73] Schadendorf D., Wolchok J.D., Hodi F.S., Chiarion-Sileni V., Gonzalez R., Rutkowski P., Grob J.J., Cowey C.L., Lao C.D., Chesney J. (2017). Efficacy and Safety Outcomes in Patients with Advanced Melanoma Who Discontinued Treatment with Nivolumab and Ipilimumab Because of Adverse Events: A Pooled Analysis of Randomized Phase II and III Trials. Clin. Oncol..

[74] Fujimura T., Sato Y., Tanita K., Kambayashi Y., Otsuka A., Fujisawa Y., Yoshino K., Matsushita S., Funakoshi T., Hata H. (2018). Serum soluble CD163 and CXCL5 could be predictive markers for immune related adverse event in patients with advanced melanoma treated with nivolumab. Oncotarget.

[75] Fujimura T., Kambayashi Y., Furudate S., Kakizaki A., Hidaka T., Haga T., Hashimoto A., Morimoto R., Aiba S. (2017). Isolated ACTH deficiency possibly caused by nivolumab in a metastatic melanoma patient. J. Dermatol..

[76] Magis Q., Gaudy-Marqueste C., Basire A., Loundou A., Malissen N., Troin L., Monestier S., Mallet S., Hesse S., Richard M.A. (2018). Diabetes and Blood Glucose Disorders Under Anti-PD1. J. Immunother..

[77] Kambayashi Y., Fujimura T., Kuroda H., Otsuka A., Irie H., Aiba S. (2020). Severe demyelinating neuropathy in an advanced melanoma patient treated with nivolumab plus ipilimumab combined therapy. Case Rep. Oncol..

[78] Yano S., Ashida K., Sakamoto R., Sakaguchi C., Ogata M., Maruyama K., Sakamoto S., Ikeda M., Ohe K., Akasu S. (2020). Human leucocyte antigen DR15, a possible predictive marker for immune checkpoint inhibitor-induced secondary adrenal insufficiency. Eur. J. Cancer.

[79] Furudate S., Fujimura T., Kambayashi Y., Kakizaki A., Aiba S. (2014). Comparison of CD163+ CD206+ M2 macrophages in the lesional skin of bullous pemphigoid and pemphigus vulgaris: The possible pathogenesis of bullous pemphigoid. Dermatology.

[80] Fuentes-Duculan J., Suárez-Fariñas M., Zaba L.C., Nograles K.E., Pierson K.C., Mitsui H., Pensabene C.A., Kzhyshkowska J., Krueger J.G., Lowes M.A. (2010). A subpopulation of CD163-positive macrophages is classically activated in psoriasis. J. Investig. Dermatol..

[81] Tojo G., Fujimura T., Kawano M., Ogasawara K., Kambayashi Y., Furudate S., Mizuashi M., Aiba S. (2013). Comparison of IL-17 producing cells in different clinical types of alopecia areata. Dermatology.

[82] Muntyanu A., Netchiporouk E., Gerstein W., Gniadecki R., Litvinov I.V. (2020). Cutaneous Immune-Related Adverse Events (irAEs) to Immune Checkpoint Inhibitors: A Dermatology Perspective on Management. J. Cutan. Med. Surg..

[83] Nelson C.A., Singer S., Chen T., Puleo A.E., Lian C.G., Wei E.X., Giobbie-Hurder A., Mostaghimi A., LeBoeuf N.R. (2020). Bullous pemphigoid after anti-PD-1 therapy: A retrospective case-control study evaluating impact on tumor response and survival outcomes. J. Am. Acad. Dermatol..

[84] Di Altobrando A., Bruni F., Alessandrini A., Starace M., Misciali C., Piraccini B.M. (2020). Severe de-novo palmoplantar and nail psoriasis complicating Nivolumab treatment for metastatic melanoma. Dermatol. Ther..

[85] Wang L., Leite de Oliveira R., Huijberts S., Huijberts S., Bosdriesz E., Pencheva N., Brunen D., Bosma A., Song J.Y., Zevenhoven J. (2018). An Acquired Vulnerability of Drug-Resistant Melanoma with Therapeutic Potential. Cells.

[86] Heijkants R., Willekens K., Schoonderwoerd M., Teunisse A., Nieveen M., Radaelli E., Hawinkels L., Marine J.C., Jochemsen A. (2017). Combined inhibition of CDK and HDAC as a promising therapeutic strategy for both cutaneous and uveal metastatic melanoma. Oncotarget.

[87] Corre S., Tardif N., Mouchet N., Leclair H.M., Boussemart L., Gautron A., Bachelot L., Perrot A., Soshilov A., Rogiers A. (2018). Sustained activation of the Aryl hydrocarbon Receptor transcription factor promotes resistance to BRAF-inhibitors in melanoma. Nat. Commun..

[88] Jenkins R.W., Barbie D.A., Flaherty K.T. (2018). Mechanisms of resistance to immune checkpoint inhibitors. Br. J. Cancer.

[89] Rozeman E.A., Menzies A.M., van Akkooi A.C.J., Adhikari C., Bierman C., van de Wiel B.A., Scolyer R.A., Krijgsman O., Sikorska K., Eriksson H. (2019). Identification of the optimal combination dosing schedule of neoadjuvant ipilimumab plus nivolumab in macroscopic stage III melanoma (OpACIN-neo): A multicentre, phase 2, randomised, controlled trial. Lancet Oncol..

[90] Ahern E., Smyth M.J., Dougall W.C., Teng M.W.L. (2018). Roles of the RANKL-RANK axis in antitumour immunity—mplications for therapy. Nat. Rev. Clin. Oncol..

[91] Ahern E., Harjunpää H., O’Donnell J.S., Allen S., Dougall W.C., Teng M.W.L., Smyth M.J. (2018). RANKL blockade improves efficacy of PD1-PD-L1 blockade or dual PD1-PD-L1 and CTLA4 blockade in mouse models of cancer. Oncoimmunology.

